# Studies of Excess Heat and Convection in a Water Calorimeter

**DOI:** 10.6028/jres.106.041

**Published:** 2001-10-01

**Authors:** John K. Domen, Steve R. Domen

**Affiliations:** Picatinny Arsenal, NJ 07806-5000; National Institute of Standards and Technology, Gaithersburg, MD 20899-0001

**Keywords:** absorbed dose, calorimeter, convection, excess heat, finite element analysis, thermistor, water calorimeter

## Abstract

To explain a difference of 0.5 % between the absorbed-dose standards of the National Institute of Standards and Technology (NIST) and the National Research Council of Canada (NRCC), Seuntjens et al. suggest the fault lies with the NIST water calorimeter being operated at 22 °C and the method with which the measurements were made. Their calculations show that this difference is due to overprediction of temperature rises of six consecutive ^60^Co radiation runs at NIST. However, the consecutive runs they refer to were merely preliminary measurements to determine the procedure for the NIST beam calibration. The beam calibration was determined from only two consecutive runs followed by water circulation to re-establish temperature equilibrium. This procedure was used for measurements on 77 days, with 32 runs per day. Convection external to the glass cylindrical detector assembly performed a beneficial role. It aided (along with conduction) in increasing the rate of excess heat transported away from the thin cylindrical wall. This decreased the rate of heat conducted toward the axially located thermistors. The other sources of excess heat are the: (1) non-water materials in the temperature probe, and (2) exothermic effect of the once-distilled water external to the cylinder. Finite-element calculations were made to determine the separate and combined effects of the excess heat sources for the afterdrift. From this analysis, extrapolation of the measured afterdrifts of two consecutive runs to mid radiation leads to an estimated over-prediction of no more than about 0.1 %. Experimental measurements contradict the calculated results of Seuntjens et al. that convective motion (a plume) originates from the thermistors operated with an electrical power dissipation as low as 0.6 μW, well below the measured threshold of 50 μW. The method used for detecting a plume was sensitive enough to measure a convective plume (if it had started) down to about the 10 μW power level. Measurements also contradict the NRCC calculations in predicting the behavior of the NIST afterdrifts.

## 1. Introduction

This paper discusses model studies and analyses to estimate systematic effects in water calorimetry for measuring absorbed radiation dosage.

The importance of measuring absorbed dose in water, *D*_w_, and its significance to radiation therapy has been stated in many publications and will not be repeated here. The reader is referred to an excellent review on water calorimetry for radiation dosimetry by Ross and Klassen [1]. Water calorimetry is based on the assumption that the temperature rise at a point in water, Δ*T*_w_, produced by radiation alone, with no transfer of heat to or from that point, is given by:

$$D_{w}=c_{w}\cdot \Delta T_{w},$$ (1)

where *c*_w_ is the specific heat capacity of water at the operating temperature of the calorimeter, and *D*_w_ is the energy per unit mass imparted to matter at a point by ionizing radiation. The SI unit of absorbed dose is the gray; 1 Gy = 1 J/kg. An absorbed dose of 1 Gy at a point in water will produce a temperature rise of approximately 0.24 mK.

Ross and Klassen [1] and their listed references also describe in detail the heat defect in radiated water, but this factor will be assumed to be unity for the purposes of the present paper. In addition, Eq. (1) assumes that Δ*T*_w_ is measured with a massless temperature probe. However, the probe is made of non-water materials that rise higher in temperature than the surrounding water, mainly due to their different specific heat capacities. The excess temperature measured by the probe changes as a function of time during and after radiation. It is only the behavior of the dissipation of the excess heat in the post radiation (afterdrift), which is taken into account in determining Δ*T*_w_.

The first part of this paper presents results of a detailed analysis of the dissipation of the excess heat in the temperature probes used to measure absorbed dose in a water calorimeter. Earlier papers [2,3] presented only a rough estimate of the upper limit of this effect as a function of time after radiation. The present paper describes the probes in minute detail, taking into account all the material properties during radiation. The probes were enclosed within a thin-wall glass cylinder. The conductive effects of the excess heat generated in the cylinder wall are also presented in more detail. Similar results were also reported by Seuntjens and Palmans [4] and by Ross et al. [5].

The second part of this paper presents a study of the effects of water convection on measurements made with the calorimeter described below. The possible existence of convection does not necessarily mean that it will have a significant adverse effect. A sensitive test is described that applies to all radiation and geometric conditions.

## 2. The Calorimeter

**Fig. 1:**
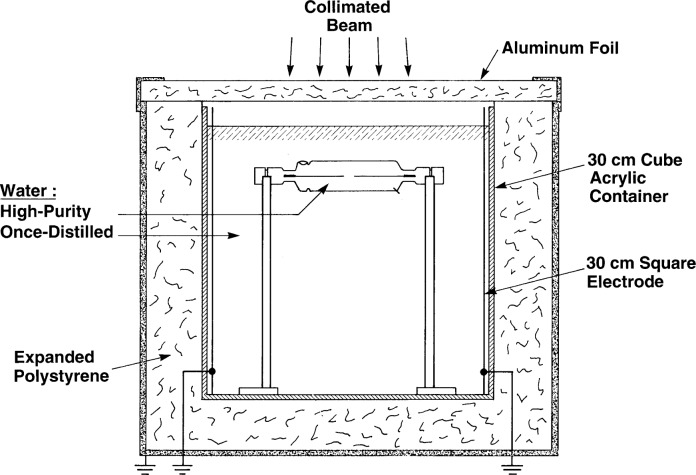
Essential features of the SW calorimeter for measuring absorbed dose.

Figure 1 shows the general features of the sealed water (SW) calorimeter radiated with a collimated ^60^Co beam directed vertically downward. The source-to-detector distance was 1 m. The size of the beam was 14.5 cm × 14.5 cm at the 50 % dose points at a measurement depth of 5 cm [6]. Details of the calorimeter and its use in measuring absorbed dose are described in reference [3].

Temperature measurements were made with two calibrated thermistor probes positioned along the axis of a thin-wall glass cylinder and connected to opposite arms of a Wheatstone bridge. The cylinder was immersed in a 30 cm cube of water surrounded by insulation. The outside diameter and wall thickness, about the central cross section of the cylinder, were 33 mm and 0.3 mm, respectively. The cylinder has a dual purpose: (1) to enclose the high-purity water, preferably saturated with high purity hydrogen, and (2) to act as a barrier to external convection that readily takes place when the calorimeter is radiated with a collimated beam. Convection (in the absence of a barrier) will cause a non-linear concave downward temperature rise, as illustrated in Sec. 8.2.

## 3. The Measurements

**Fig. 2:**
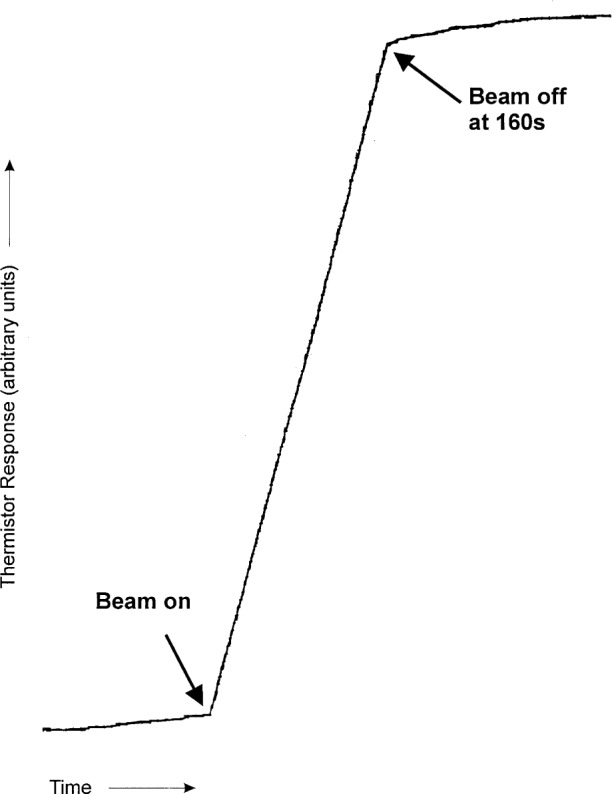
Radiation run showing linearity of response and initial and post radiation drifts.

The effectiveness of the cylinder as a barrier to external convection is shown by the recording in Fig. 2. The thermistor powers were approximately 100 μW. The temperature rise is linear during the 160 s of radiation, and the initial part of the afterdrift appears to be the same as the initial drift. The latter part of the afterdrift begins to show a slight decrease in slope at about 40 s after beam turnoff. The beginning of this cooling trend appears to be the result of conduction within the cylinder caused by external convection that transports cooler water to the vicinity of the cylinder (also discussed in Sec. 8.2). In the absence of external convection, the cooling trend would have been noticeable at a much later time (see Fig. 1 of [7]) as a result of conduction starting mainly in the beam penumbra. The behavior of the afterdrift shown in Fig. 2 contradicts the calculated predictions of Seuntjens et al. [8]. Their Fig. 20 and Table 4 predict a rapid rate of decrease in the afterdrift at the 100 μW level for a radiation time of only 70 s.

**Fig. 3:**
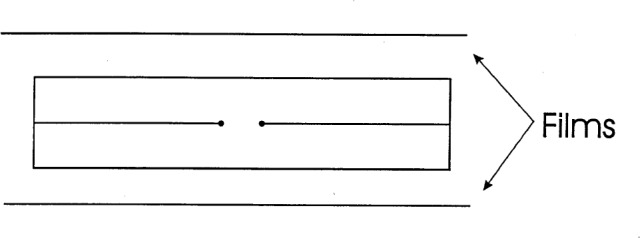
Sketch of detector assembly and protective film barriers against external convection.

A design to reduce this convective effect is shown in Fig. 3, which shows thin films mounted above and below the cylinder. These would have a dual role: (1) to shield the cylinder from external convection during radiation, and (2) to act as a brake to quickly stop the water motion after circulation. This would cause the initial drift to steadily decrease and be more predictable. The films were not in position when the reported measurements were made [3].

**Fig. 4:**
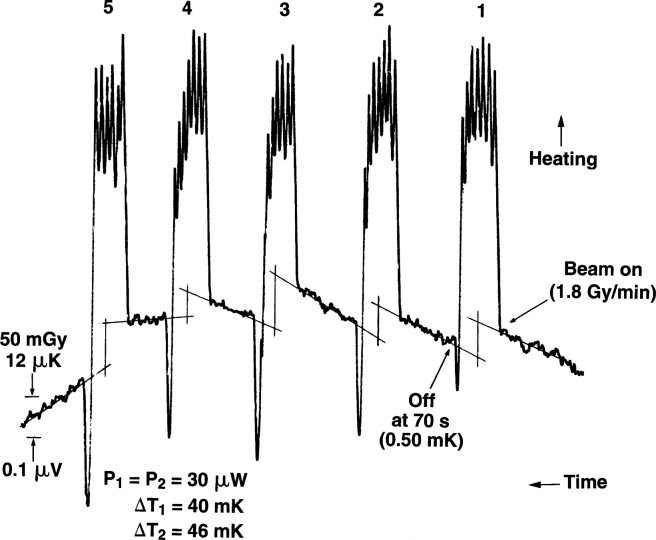
Typical preliminary set of consecutive runs. Time increases from right to left. The heating is caused by the thermistor response to absorbed radiation. The spikes are caused by the heating and manual adjustments of the Wheatstone bridge balancing arm to keep the recorder trace on scale.

Figure 4 shows the temperature traces of a typical series of five consecutive runs. Time increases from right to left. The spikes shown are caused by radiation heating and manual adjustments of the Wheatstone-bridge balancing arm to keep the recorder trace on scale. The duration of the runs was varied around 70 s to produce a temperature rise of 0.5 mK. Observation of these preliminary runs was helpful in determining the measurement procedure for the beam calibration. It was observed that the drifts of the first two runs are essentially constant, while the afterdrift of the third run shows a small change as a result of a cooling effect that increased, as indicated, in subsequent drifts. This was caused by the external convective cooling effect that eventually reached the thermistor by internal conduction sometime during the third radiation run. Therefore, the measurement procedure was to make only two consecutive runs followed by circulation of the once-distilled water. This pattern of runs was made on 77 days in which 32 runs were made on each day. It required about 6 h to make these runs, including the time to fill the water container to an exact level each day, circulate the water, and reduce the drift to an acceptably small value. Although the radiation runs were about 70 s, calculated studies of excess heat caused by the probes are presented in Sec. 6.1 for radiation runs from 30 s to 180 s.

## 4. The Probes

The most important design consideration in fabricating the probes is to limit the mass of materials to minimize the excess heat generation during radiation, while still maintaining sufficient sturdiness of the long thin probes. The thermistor is sealed in a Pyrex^1^ glass capillary that had an outside diameter close to 0.4 mm and an inside diameter of 0.3 mm. This provided sufficient sturdiness as well as a practical limit to those small dimensions. Those dimensions were achieved by drawing down a larger dimensional capillary heated with a flame [3].

**Fig. 5:**
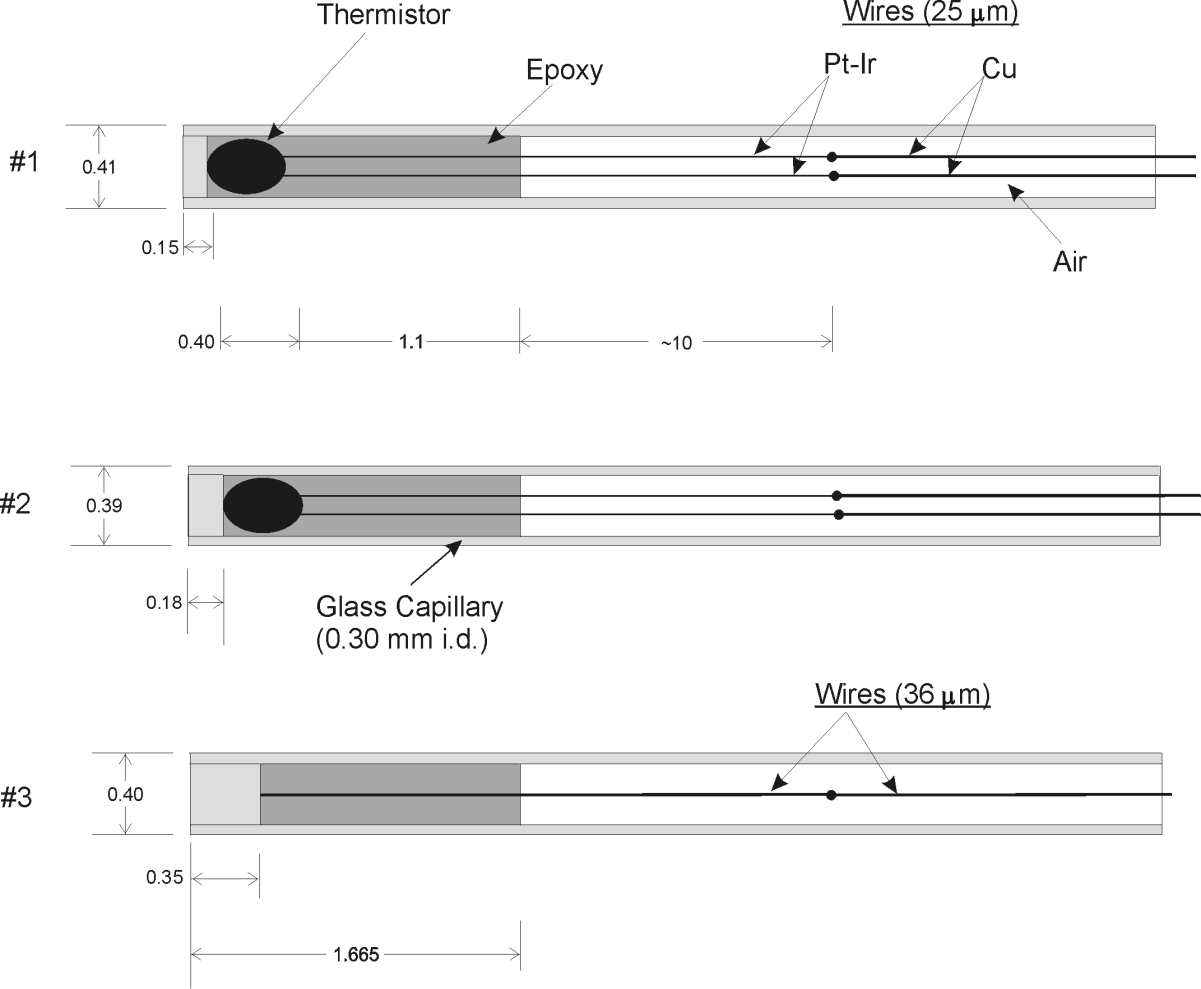
Details of measurement probes #1 and #2 and computer-model probe #3. Dimensions are in millimeters.

Figure 5 shows details of probes #1 and #2. The optically determined dimensions shown are in millimeters. The thermistors were embedded in epoxy to provide good thermal contact with the capillaries. The mass of epoxy was kept small by limiting its length to about 1.1 mm (as shown) beyond the thermistors in the capillary. The capillary end was enclosed with a tapered glass plug ground down to a thickness of either 0.15 mm or 0.18 mm, which gives an average thickness of 0.165 mm. A computer analysis of the excess heat behavior took into account all the dimensions, and values of the physical, thermal, and radiation properties of the materials shown. The values are listed in Table 1.

A somewhat simplified computer model of the probes is illustrated in Fig. 5 as probe #3. Its diameter is 0.40 mm, the average of probes #1 and #2. The glass coated bead thermistors approximate the shape of a pro-late spheroid, when viewed with a microscope, with major and minor axes of 0.40 mm and 0.25 mm, respectively. Its calculated volume is 6 % of the sensor zone, extending from the tip of the probe to the epoxy-air boundary. It appeared that at least half of the thermistor volume was glass. For simplification the thermistor was assumed to be all glass. A cylinder with a diameter of 0.30 mm, and thickness of 0.185 mm, would fit within the capillary and have a volume equal to that of the thermistor. The glass plug plus the cylinder results in a thickness of 0.350 mm of glass for probe #3. The end position of the wire (90 % Pt and 10 % Ir) is taken to be at this position and fused to the glass. This is close to the actual center of the thermistor, (0.165 + 0.20) mm = 0.365 mm. The length of the sensor zone remains unchanged, (0.165 + 0.40 + 1.1) mm = 1.665 mm.

**Fig. 6:**
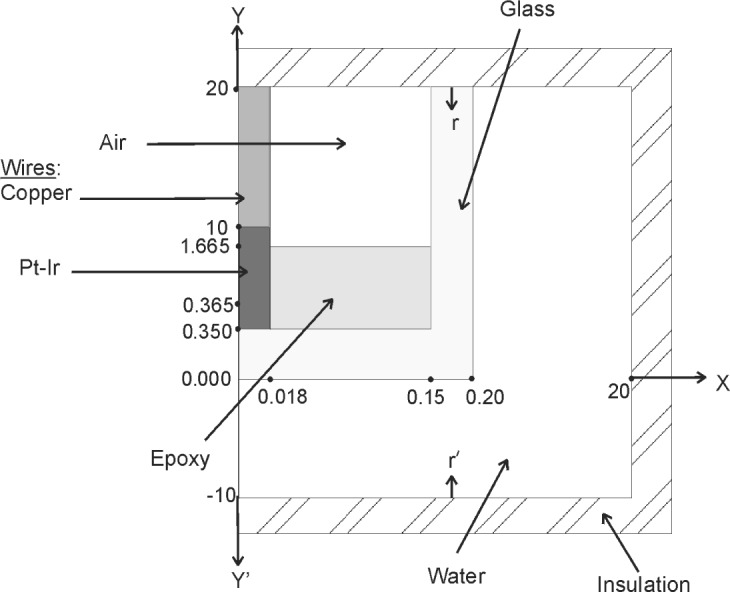
Further details of computer model of probe #3 immersed in water (not to scale). Dimensions are in millimeters.

To facilitate computation in the computer model, the 25 μm diameter wires were replaced with a single wire 36 μm in diameter, along the central axis of the capillary. Figure 6 shows an expanded dimensional drawing (not to scale) through the central axis of probe #3 in a vertical position and immersed in water surrounded by impervious heat boundaries. The assumed radius of the high purity water bath is 20 mm and the length is 30 mm. The radius is sufficiently large so that no excess heat reached its boundary for the longest radiation run (180 s) studied. The tip of the probe is along the *x*-axis. The axis through the center of the wall thickness (at *x* = 0.175 mm) is *rr*′, extending from the water boundaries from *y* = −10 mm to 20 mm.

## 5. Computer Analysis

The time-dependent thermal solution was obtained with the ANSYS 5.6 finite-element engineering software. Almost 120 000 axisymmetric thermal solid finite ring elements type Plane55 intersect the plane from −10 mm to 20 mm in the *y* direction, and 0 mm to 20 mm in the *x* direction. These elements are applicable for transient thermal analysis for conduction, internal heat generation, and film coefficient convective flow. Four elements were used for the radial thickness of the probe sidewall, ten for the epoxy and for air, and three for the wire. A greater radial meshing was not required; when tested, it resulted in a change in the excess temperature of only about 10^−4^ %. The heat generation rate *Q* (W/cm^3^) in the calculation was determined by

$$Q=c\;d\;t_{w}\;R$$ (2)

where for each of the six materials, *c* is the specific heat capacity, *d* is the density, *t*_w_ is the experimental temperature rise of water in one second (0.0005/70 °C/s), and *R* is the relative temperature rise of each material to that of water for 1 MeV photons.

The thermal solution for every node location was determined for the entire time from the start of radiation through the post radiation. During radiation the integration times were a few seconds. In the interval of 2 s before beam turn off, the integration times were reduced from 0.1 s to 0.05 s. In the interval of 2 s after beam turn off, the integration times were increased from 0.05 s to 0.1 s. The longest radiation studied was 180 s followed by 120 s of post radiation. Excess heat did not arrive at the far adiabatic boundary, as indicated by the constancy of six-figure temperature printouts from the time of the beam turn off to the end of the post radiation.

## 6. Excess Heat

In this section we discuss the models of excess heat caused by the probes, the cylinder wall and the once-distilled water.

### 6.1 Probes

Table 2 lists the calculated excess thermistor temperatures as a function of time after radiation, from 0 s to 60 s, for radiation times from 30 s to 180 s. The results show that the excess heat reduces to small values after about 5 s. The nominal measurement radiation times were 70 s, and therefore will be emphasized through the remainder of this paper.

**Fig. 7:**
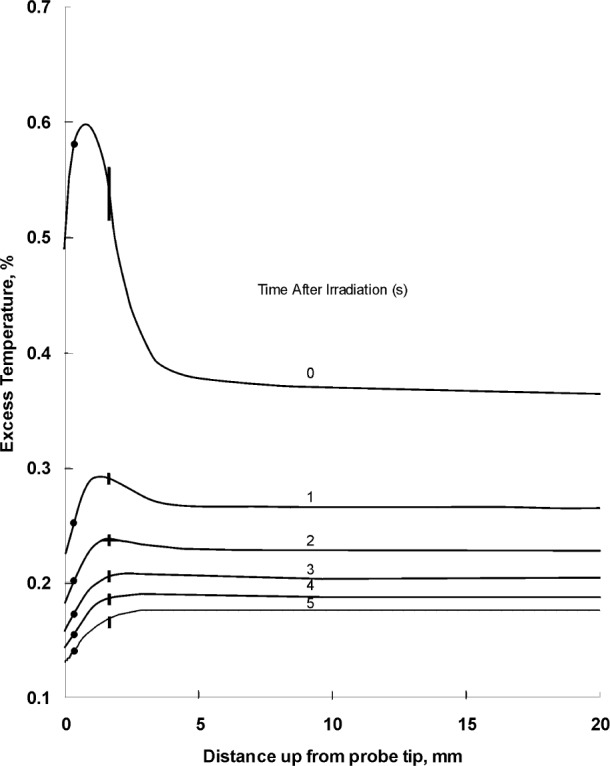
Excess temperature along the probe axis as a function of time after a 70 s radiation run. The dots and vertical lines are, respectively, the positions of the thermistor and sensor zone boundary.

**Fig. 8:**
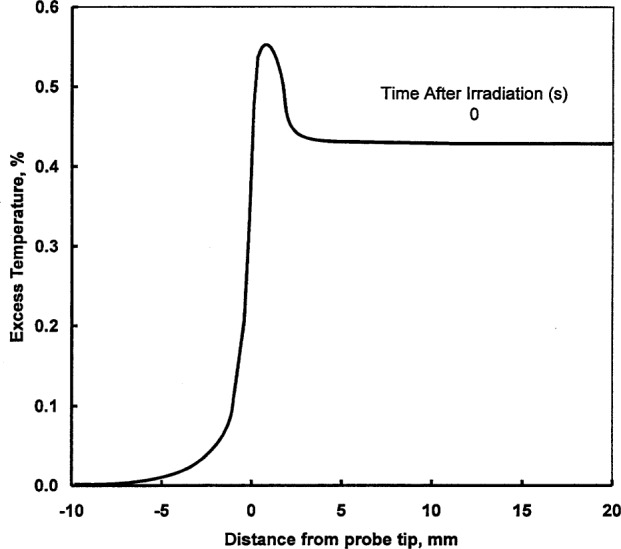
Excess temperature along *rr*′ (at *x* = 0.175 mm), through the center of the capillary wall after a 70 s radiation run.

Table 3 lists the calculated excess thermistor temperatures for probe diameters from 0.4 mm to 1.0 mm. Figure 7 shows the axial distribution of excess temperature after a 70 s radiation for the 0.4 mm diameter probe. The dots and the vertical lines on the curves represent, respectively, the positions of the thermistor (at *y* = 0.365 mm) and the sensor zone boundary (at *y* = 1.665 mm), as given in Fig.6. Figure 8 shows the excess heat distribution, at beam turn off, along *rr*′ (Fig. 6 at *x* = 0.175 mm), through the center of the capillary wall. The decrease of excess heat beyond *y* = 1.665 mm is a result of heat being extracted along the capillary, from the sensor zone. It is then radially conducted to the water and essentially vanishes at about 3 mm beyond the sensor zone.

An inspection of the plateaus, at 0 s in Fig. 7 and Fig. 8, shows that the capillary wall is 0.06 % higher in temperature than along the capillary central axis. Similar calculations (not shown) as in Fig. 8 were made for post radiation times from 1 s to 5 s. The results showed that the plateaus exactly coincided with those along the central axis shown in Fig. 7. Computer runs were also made with the Pt-Ir and copper wires removed. The calculations showed that the wires contributed less than 0.01 % in extracting excess heat from the sensor zone.

### 6.2 Cylinder Wall

**Fig. 9:**
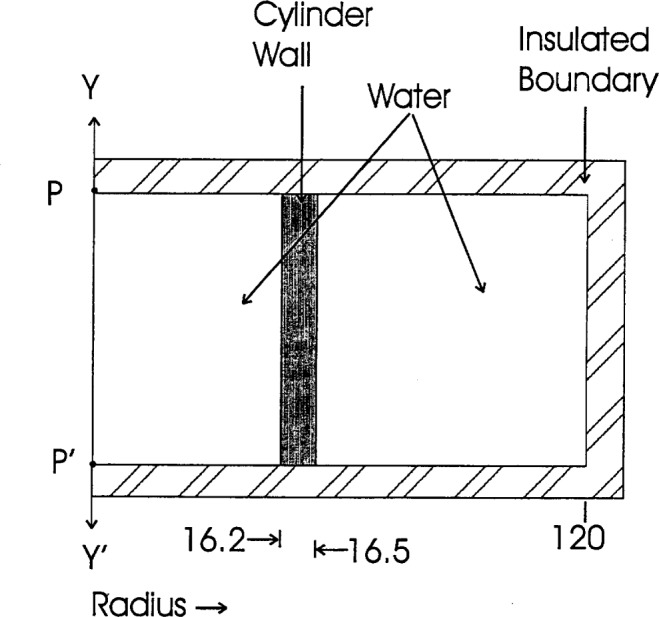
Sketch of computer geometry used for calculating conductive heat flow from the cylinder wall. Dimensions are in millimeters.

**Fig. 10:**
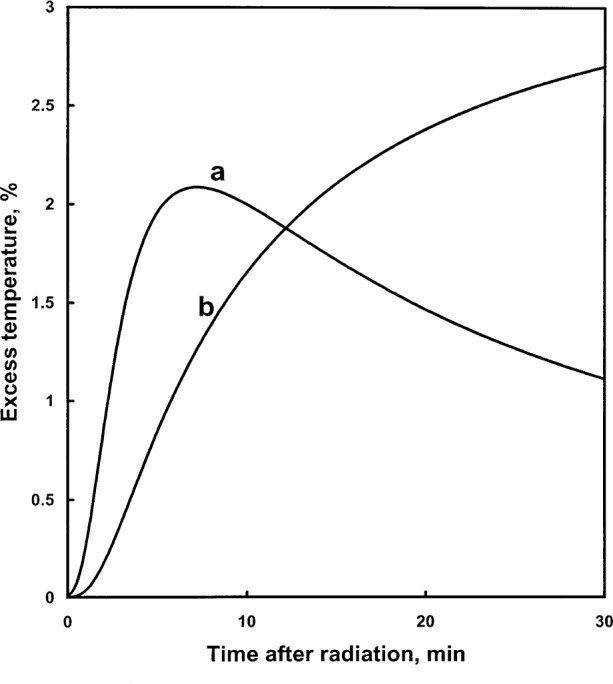
Conductive contributions to excess thermistor temperature from the cylinder glass wall (curve a) and exothermic effect from the external once-distilled water (curve b) after 70 s of irradiation.

Figure 9 shows the geometry selected for the computer calculations. The glass wall was assumed to be uniform in thickness (0.3 mm) and infinite in extent so that the calculated flow of heat is perpendicular to the glass wall. Therefore, the distance *PP*′ along the cylinder and probe axis (*YY*′) could be chosen to be any convenient length. An impervious heat boundary was chosen to be at 120 mm, large enough that the annular water ring, radii of 16.5 mm to 120 mm, would act as an infinite heat sink for excess heat conducted from the cylinder wall such that no excess heat would arrive at the boundary 30 min after 70 s of radiation. Figure 10 (curve a) shows the calculated excess thermistor temperature as a result of excess heat arriving from the glass wall. The excess heat, along the axis, is at a maximum at 498 s after radiation. Table 4 shows the percent excess values from 0 s to 100 s.

### 6.3 Once-Distilled Water

The once-distilled water external to the cylinder (see Fig. 1) was previously found to have a measured exothermic effect of 3.5 % during radiation [7]. The internal high-purity water, saturated with high-purity hydrogen, is assumed to have a zero heat defect. Therefore, the relative rates of temperature rises internal and external to the cylinder are 1 to 1.035, respectively. The region between radii 16.5 mm and 120 mm (see Fig. 9) simulates an infinite source of excess heat conducted radially toward the thermistors located along the central axis, *YY*′. The calculated results, after 70 s of radiation, are plotted in Fig. 10 (curve b).

## 7. Combined Effects

**Fig. 11:**
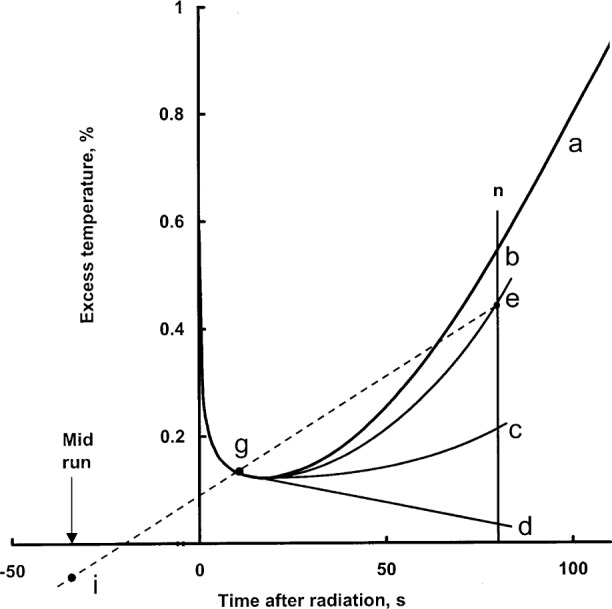
Illustration of excess temperature afterdrifts after 70 s of radiation. Curve a is the combined conductive effect. Curves b, c, and d are subjectively drawn to illustrate the convective effect on the afterdrifts of runs 1, 2, and 3, respectively. Line n is at 80 s, the approximate duration of the afterdrifts. Extrapolation points are shown as e, g, and i (at −35 s, the mid-run).

Table 4 lists the separate and combined effects. Figure 11, curve a, is a plot of the combined effects. It is the conductive afterdrift of the first run. Section 9 describes an approximate method for making a correction for a non-linear drift. But the calorimeter was operated at room temperature where buoyant forces causing convection can easily arise and the films shown in Fig. 3 were not in position. Therefore, convection external to the cylinder and its effect on the calculated afterdrift must be taken into account before considering the extrapolation to the midrun to correct for excess heat effects.

## 8. Convection

Temperature gradients are a source of convection when they produce buoyant forces strong enough to overcome the opposing force of the viscous drag of the fluid. Two sources of temperature gradients of interest here are: (1) those closely confined around the thermistors because of their electrical heating, and (2) those produced by radiation, particularly with collimated beams.

### 8.1 Thermistor Gradients

It is initially assumed that the thermistor consists of a bare isolated sphere, 0.25 mm in diameter, immersed in and at the same temperature as the stagnant water. When the thermistor power is increased from zero to some moderate value, temperature gradients will be set up near its surface, but the water will not be set in motion. Some further increase in power will cause the water near the surface to move, but still be confined to that region because of the opposing viscous force of the fluid. This confined circulation is in temperature equilibrium and should have no effect on measurements of absorbed dose. When the thermistor heating power is further increased, there will be a certain power at which the buoyant force is greater than the viscous drag. A convective plume will begin to form and will increase rapidly in size as the power is increased. The onset of the convective plume was observed to start at about 50 μW with an immersible thermistor [9]. The detailed construction is described in Sec. 7 of reference [9]. There was less non-water material in the sensor zone than that shown in Fig. 5. The immersible thermistor was not embedded in a glass capillary, which increases the sensor diameter and contributes to extracting heat from the sensor zone along the capillary wall as illustrated in Fig. 8, particularly at the instant of beam turn off (i.e., at 0 s). The sharp temperature gradient beyond the sensor zone boundary (shown by the vertical lines of Fig. 7) is caused by heat extracted from that zone. Therefore, it appears reasonable to conclude that, for glass capillary temperature probes, the onset of a convective plume will occur at some power above 50 μW. The onset power will depend on the detailed physical structure, and will increase as the probe diameter is increased.

### 8.2 Radiation Gradients

**Fig. 12:**
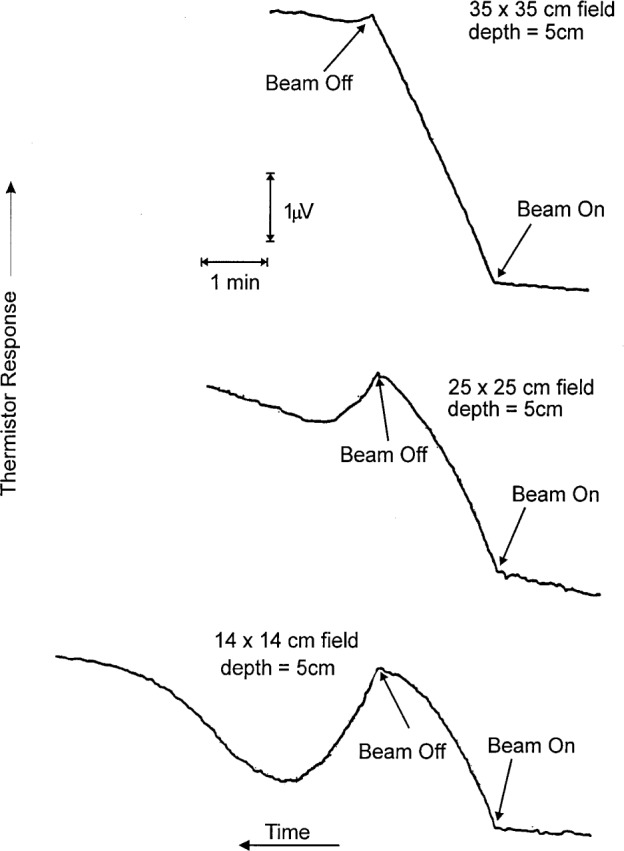
Recordings of thermistor circuit response as a function of radiation field size. Time increases from right to left. Reproduced with permission of R. B. Barnett.

Figure 12 shows the effects of convection with an immersible thermistor with no convective barrier in its vicinity [10]. The radiation conditions were similar to those of the present experiment, mentioned in Sec. 2. The ^60^Co beam was directed vertically downward, and the measurement depth was also at 5 cm, at a source-to-detector distance of 80 cm. Figure 12 shows large variations in response when the field size is varied [10]. The 14 cm × 14 cm field is almost identical to the 14.5 cm × 14.5 cm field of the present experiment. It was pointed out [10] that the convection was caused by large temperature gradients in the beam penumbra. By using these measurements and the results of another experimental investigation [9], it is possible to determine the convective velocity at the thermistor.

**Fig. 13:**
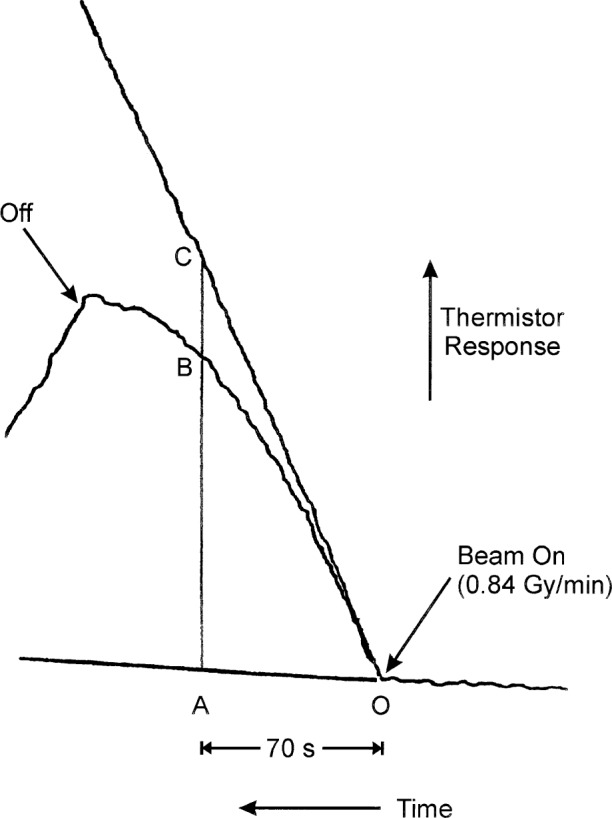
Relative responses of recordings of Fig. 12 with 14 cm × 14 cm and 35 cm × 35 cm field sizes, after superposition of their initial drifts.

Figure 13 is a response comparison with the 14 cm × 14 cm and the 35 cm × 35 cm fields, when their initial drifts are made to coincide. The temperature rise in the larger field is almost linear up to the end of the 2 min radiation. The non-linear rise with the smaller field is a result of convective cooling of the thermistor, equivalent to a “negative” absorbed dose rate [9]. The thermistor electrical power was 25 μW, and the absorbed dose rate was 0.84 Gy/min. The time from 0 to A is 70 s. It is desired to determine both the average convective velocity during a radiation period of 70 s and the velocity at beam turn off, after a radiation time of 70 s. The first step in determining the former quantity is to determine the ratios of area zones OBC to OAC. Determining the latter quantity requires the ratio of distances BC/AC.

It was convenient to determine the ratio of the areas from the ratio of measured equivalent *masses*. Photocopy enlargements were made of Fig. 13. Then paper cutouts were made of zones OAC and OBC and weighed. The results are listed in Table 5. The average negative absorbed dose rate is: (0.1650) (0.84 Gy/min) = 0.139 Gy/min.

**Fig. 14:**
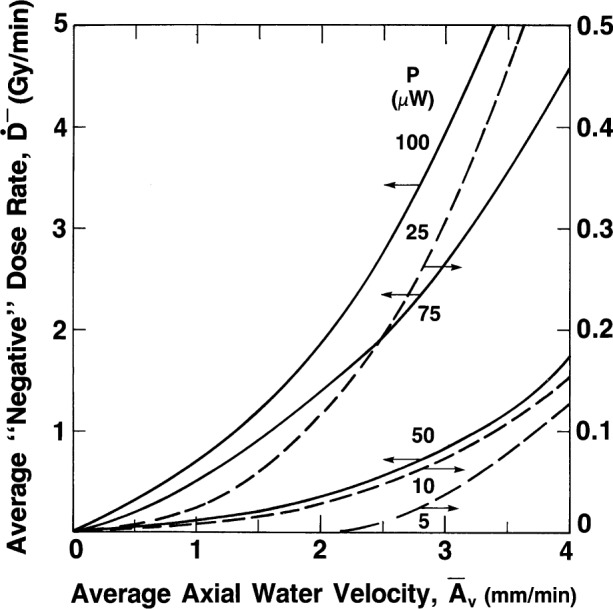
Measurements of average “negative” absorbed dose rates, *D*^−^, as a function of the average water velocity.

We then estimate the effective water velocity from this dose rate as follows. Figure 14 is a plot of a study of the convective velocity effects on a thermistor in water [9]. The thermistor used was the same immersible type as that used for the measurements shown in Fig. 12 [11]. Applying Fig. 14 to the values of 0.139 Gy/min and noting that the thermistor power was 25 μW, the average convective velocity was estimated to be 2.16 mm/min. However, the absorbed dose rate for the present experiment was 1.8 Gy/min (Fig. 4). Because the dose rate is also the driving force causing convection, the average convective velocity at the position of the thermistors (Fig. 1) with no convective barrier is estimated to be (2.16 mm/min) (1.8 Gy/min) / (0.84 Gy/min) ≅ 5 mm/min.

By using the ratio BC/AC and applying the above procedure, the convective velocity at that position (70 s after the start of radiation) was estimated to be approximately 6 mm/min. Placement of the cylinder as shown in Fig. 1 would cause the convective velocities within the region of the detector assembly to essentially vanish. It appears unlikely that for smaller collimated beams with varying dose profiles and dose rates of several Gy/min that internal velocities would amount to several mm/min. Even in this unlikely case, studies with forced convection showed that a convective velocity of 2.5 mm/min on a thermistor at 5 μW of power produced a cooling effect on its equilibrium temperature that was barely detectable above the noise level [9].

The recordings shown in Fig. 12 and the measured results shown in Fig. 14 were determined, as previously mentioned, with the same type of thermistor. When immersed in water, they had a measured equilibrium temperature rise of 1.41 mK/μW of electrical power dissipation [9]. Therefore, prior to the radiation runs shown in Fig. 12, the equilibrium temperature rise is estimated to be (1.41 mK/μW) (25 μW) = 35.3 mK. For a 70 s radiation run, a linear temperature rise (such as with the 35 cm × 35 cm field) would result in a temperature rise at beam off of only (0.84 Gy/min) (1 min/60 s) (70 s) (0.24 mK/Gy) = 0.24 mK.

This is only a 0.7 % temperature rise above the initial equilibrium temperature. Therefore, slight convective cooling disturbances of the background equilibrium temperature rise of the thermistor can result in significant departures from linearity as shown in Fig. 12. An examination of the plots shown in Fig. 14 shows that the sensitivity of detecting a convective disturbance rapidly increases with thermistor power. There is roughly an order of magnitude increase in sensitivity in detecting convection when the thermistor power is increased from 25 μW to 100 μW.

For the run shown in Fig. 2, the thermistor powers of the two thermistors (enclosed in the cylinder) were 98 μW and 102 μW. The rise of that run does not show the slightest concave downward evidence of internal convection such as that shown in Fig. 12 with the 25 cm × 25 cm and with the 14 cm × 14 cm fields. The absorbed dose measurements, in the present experiment (Fig. 4), were made with thermistor powers of 30 μW. Therefore, internal convection was either non-existent or any microscopic effects at the 100 μW level (Fig. 2) were further reduced to insignificance when the thermistor powers were reduced to 30 μW as used for the absorbed dose measurements [3]. The measurements showed no significant change when the thermistor powers were varied from 9 μW to 100 μW [3]. Krauss and Roos [12] arrive at a similar conclusion for horizontal ^60^Co radiation when the thermistor powers were varied from 15 μW to 120 μW in a 40 mm diameter glass cylinder.

The decreasing-then-increasing behavior of the afterdrift shown in Fig. 12, particularly with the 14 cm × 14 cm field, was mainly the result of a gradual diminishing of the convective velocity when its driving force was terminated (beam turn-off). This behavior was not the result of excessive inertia of the thermistor in its rise to a new temperature level, because if the convective velocity had instantly vanished the thermistor would have rapidly reestablished its new equilibrium temperature. Two sets of measurements were made to determine the behavior of the thermistor when subject to an instantaneous change of power.

The first test was performed with the earlier version of the calorimeter in which the thermistors were sandwiched between two polyethylene films. The results showed that, when a sudden change in thermistor power was applied, about 90 % of the change to its new equilibrium temperature was established after 2 s, and about 97 % after 5 s (see the recording in Fig. 14 of [7]). The second test was done with probes #1 and #2. The results are given in Table 6. The data indicate that there might be a real, but small, difference in response depending whether the power was increased (I) or decreased (D). Because nominal results are sufficient for this demonstration, the values shown are averaged. The results show that at 2 s and at 5 s after a sudden power change, the response is 91 % and 95 %, respectively, of the change to a new equilibrium temperature. The two tests are thus in good agreement, although the geometries are different. These measured results are in contradiction to the calculated results of Seuntjens et al. [8]. Their Fig. 5 shows that it takes 100 s to reach about 97 % of the new equilibrium temperature.

From the above analysis, and by referral to Fig. 4, the external convective velocity had not vanished to zero when the second radiation run began. Therefore, for the five consecutive runs shown, the convective velocities gradually increased, during and after radiation causing the cooler water at a greater depth to be drawn more rapidly upward near the surface of the cylinder. This resulted in internal conduction and an eventual reversal of the remaining internal excess heat flow and then to cause the increasing cooling drifts shown. Note that Fig. 2 shows no upward curvature in the measured afterdrift, which would be predicted in the absence of convection as shown in Fig. 11.

## 9. Extrapolation

Extrapolation as that shown in Fig. 11 is a procedure which may require subjective judgment. The usual way of treating a non-linear afterdrift is first to ignore the duration of any observable sharp drop in temperature after the beam is turned off [4, 5, 13]. This is not consistently observable in the present case (Fig. 4), because of the relatively small amounts of non-water materials in the sensor zones of probes #1 and #2. When small short-duration overshoots were observed at beam-off, they were ignored in the extrapolations. Other causes of such overshoots could have been noise signals and magnetic pickup when the beam shutter was closed, even though magnetic shielding was provided for most of the set-up.

The calculated results shown in Fig. 11, curve a, are based on the assumption that conduction was the only mode of heat transfer in stagnant water. This would be the case when the calorimeter is operated at 4 °C [13], and it would be nearly true when the calorimeter is operated at room temperature with the protective film barriers shown in Fig. 3. In these cases, a method of correcting for the excess heat is to make an empirical fit to curve a, subtract this signal from the measured post radiation, and then extrapolate the remaining main signal. Such a proposal for correcting excess heat effects was made in a previous paper [3].

But the protective films were not in position. Figure 13 shows that convection in the vicinity of the cylinder started almost immediately after beam turn-on. Therefore, convection had a significant effect in increasing the rate of excess heat transferred outside and away from the wall and decreasing the heat conducted toward the thermistors. This would decrease the slope and curvature of the first run afterdrift, illustrated by curve b in Fig. 11 (this is subjectively drawn). It is reasonable to expect that the second run afterdrift would have even less curvature, curve c, (also subjectively drawn) and would be significantly lower than curve b. At some time during the third radiation of Fig. 4, heat begins to flow from the thermistors toward the wall. This is indicated by a small but noticeable decrease in slope (cooling) as shown in the third afterdrift, Fig. 4, and illustrated as curve d in Fig. 11. Curves b and c represent transitional curves between the upper and lower limits of curves a and d, which in reality must have occurred.

Shown in Fig. 11 is a vertical line, n, at 80 s, which was approximately the average duration of the afterdrifts. Line n intersects curve b at point e. Point g was chosen at 10 s after beam off when most of the excess heat from the probe dissipated. Drawing a straight line from point e through g gives point i at the mid-run, a slight under prediction of the excess temperature there.

Curve b also represents the initial drift of the second run, while curve c represents its afterdrift. Consideration of these curves with the second radiation, not shown, appears would result in a small overprediction of the excess temperature at the mid-run. It is concluded that the extrapolation of the drifts of the first two runs (Fig. 4) to the mid-run (Fig. 11) would result in an estimated overprediction of no more than about 0.1 % due to the combined effects of the probes, cylinder wall, and exothermic effect of the external water.

Others may derive different results when determining a method and drift curves for extrapolating to the mid-run, but it appears that the differences would be small. Therefore the conclusion of this analysis is in disagreement with the claim of Seuntjens et al [8] that convection and heat transfer from the cylinder wall of the NIST calorimeter leads to a 0.5 % overprediction in the measured temperature rise (i.e., the extrapolated value at mid-run).

## 10. Conclusions

It is desirable to use only minimal amounts of non-water materials for the sensing zones of the temperature probes, such as shown in Fig. 5. The practical minimal dimensions of the zones are 0.40 mm outside diameter and about 1.7 mm long. It was pointed out [3] that for room temperature operation, the cylinder inside diameter should be perhaps not larger than 35 mm. The onset of convection is determined by the Rayleigh number, which varies with the cube of a geometric dimension. Therefore, significant effects of convection will eventually be observed as the cylinder diameter is increased. An essential test for significant internal convection is to measure the absorbed dose rate as a function of thermistor power [3]. The sensitivity of this test increases rapidly as the thermistor power is increased, and if there is no observable change, then there are no significant *effects* of convection (or they may have been non-existent) as a result of the radial and longitudinal beam profile. If there is an observable change at high powers, then the thermistor powers should be reduced to within the range where effects had not yet set in.

## 11. Summary

The effects of excess heat produced in a detector assembly, consisting of thin glass temperature probes within a thin-wall glass cylinder, used for reported measurements [3] have been studied in detail. Sensitive measurements showed that there was no internal convection within the cylinder when the water calorimeter was radiated with a collimated ^60^Co beam directed vertically downward. External convection readily set in because of large temperature gradients in the beam penumbra where the water flow was uninhibited due to a lack of convective barriers outside the cylinder. The external convection significantly aided in transporting the excess heat away from the cylinder wall, resulting in a significant reduction of excess heat conducted toward the probes located along the cylinder axis.

## Figures and Tables

**Table 1:** Material parameters affecting excess heat

Material	Density	Specific heat capacity	Thermal conductivity	*μ*_en_/*ρ*^a^	Normalized rate of energy input per unit volume
	(kg/m^3^)	[J/(kg·K)]	[W/m·K)]	(m^2^/kg)	
Water	1000	4181	0.602	0.03103	1.0
Pyrex	2230	837	1.130	0.02774	1.99
Epoxy	1200	1423	0.665	0.03015^b^	1.17
Copper	8900	385	385	0.02562	7.35
Pt(90 %)-Ir(10 %)	21600	134	31	0.03454	24.0
Air	1.2	1004	0.023	0.02789	0.001

a Mass energy absorption coefficients for 1 MeV photons.

b Value for polymethyl methacrylate.

**Table 2:** Excess thermistor temperature, %, probe diameter 0.4 mm

Radiation time (s)	Time after radiation (s)										
	0	0.1	0.2	0.5	1	2	5	10	15	30	60
30	1.23	0.91	0.80	0.63	0.50	0.38	0.25	0.17	0.13	0.07	0.04
60	0.65	0.49	0.44	0.35	0.29	0.23	0.16	0.11	0.09	0.06	0.03
70	0.56	0.43	0.38	0.31	0.25	0.20	0.14	0.10	0.08	0.05	0.03
90	0.45	0.34	0.30	0.25	0.20	0.16	0.12	0.09	0.07	0.05	0.03
120	0.34	0.26	0.23	0.19	0.16	0.13	0.10	0.07	0.06	0.04	0.03
150	0.28	0.22	0.19	0.16	0.13	0.11	0.08	0.06	0.05	0.04	0.02
180	0.23	0.18	0.16	0.13	0.11	0.09	0.07	0.05	0.04	0.03	0.02

**Table 3:** Excess thermistor temperature after a 70 s radiation, %

Probe diam. (mm)	Time after radiation (s)											
	0	0.1	0.2	0.5	1	2	5	10	15	30	60	90
0.4	0.56	0.43	0.38	0.31	0.25	0.20	0.14	0.10	0.08	0.05	0.03	0.02
0.5	0.91	0.73	0.66	0.54	0.46	0.37	0.27	0.20	0.16	0.11	0.07	0.05
0.6	1.29	1.07	0.97	0.82	0.70	0.58	0.43	0.32	0.26	0.18	0.11	0.08
0.7	1.71	1.46	1.33	1.13	0.98	0.82	0.61	0.46	0.38	0.26	0.16	0.12
0.8	2.16	1.88	1.73	1.48	1.29	1.08	0.81	0.62	0.51	0.35	0.22	0.16
0.9	2.63	2.33	2.16	1.87	1.63	1.38	1.04	0.79	0.66	0.45	0.29	0.21
1.0	3.14	2.82	2.62	2.28	2.00	1.70	1.30	0.99	0.83	0.57	0.36	0.26

**Table 4:** Excess thermistor temperature caused by probe, wall, and external H_2_O, % × 10^2^

Cause	Time after 70 s radiation (s)										
	0	10	20	30	40	50	60	70	80	90	100
Probe	56	10	6.9	5.4	4.4	3.8	3.3	2.9	2.6	2.3	2.1
Wall	1.3	2.7	5.0	8.4	13	19	27	36	45	55	66
External H_2_O	0.1	0.2	0.4	0.8	1.4	2.3	3.5	5.0	6.9	9.1	12
Total	57	13	12	15	19	25	34	44	55	66	80

**Table 5:** Relative data on heating and cooling zones

Sample	Relative mass of zone (mg)		Ratio
	OAC	OBC	OBC/OAC
1	199.95	33.68	0.1684
2	203.38	33.16	0.1630
3	207.62	34.23	0.1649
4	204.71	34.54	0.1687
5	205.15	32.84	0.1601
Average			0.1650

**Table 6:** Response of probes to sudden power changes

Probe number	Test number	Response to equilibrium, % Time after change		Power^a^
		2 s	5 s	
1	1	88.3	93.4	I
1	2	91.1	95.0	D
1	3	88.4	93.6	I
1	4	91.1	95.0	D
2	5	89.8	94.2	I
2	6	92.0	95.5	D
2	7	92.4	95.0	I
2	8	91.8	95.4	D
Average		91	95	

a I: Increased; D: Decreased.

## Footnotes

1 Certain commercial equipment, instruments, or materials are identified in this paper to foster understanding. Such identification does not imply recommendation or endorsement by the National Institute of Standards and Technology, nor does it imply that the materials or equipment identified are necessarily the best available for the purpose.
